# Supplementary material for: Effects of Icariin and Its Metabolites on GPCR Regulation and MK-801-Induced Schizophrenia-Like Behaviors in Mice
Source: Molecules. 2023 Oct 27;28(21):7300. doi: 10.3390/molecules28217300 (PMC10647531; doi:10.3390/molecules28217300)
Supplement: Supplementary file 1 [file molecules-28-07300-s001.zip › molecules-2646190-supplementary.pdf]

Supplementary Materials

Effects of Icariin and Its Metabolites on GPCR Regulation and MK-801-Induced Schizophrenia-like Behaviors in Mice

Su Hui Seong [1], Seo Hyun Kim [2], Jong Hoon Ryu [3], Jin-Woo Jeong [1], Hyun Ah Jung [4,*] and Jae Sue Choi [5,*]

[1] Division of Natural Products Research, Honam National Institute of Biological Resources, Mokpo 58762, Republic of Korea; shseong@hnibr.re.kr (S.H.S.) jwjeong@hnibr.re.kr (J.-W.J.)
[2] Division of Research Management, Honam National Institute of Biological Resources, Mokpo 58762, Republic of Korea; kshgg0808@hnibr.re.kr (S.H.K.)
[3] Department of Biomedical and Pharmaceutical Sciences, Kyung Hee University, Seoul 02447, Republic of Korea; jhryu63@khu.ac.kr (J.H.R.)
[4] Department of Food Science and Human Nutrition, Jeonbuk National University, Jeonju 54896, Republic of Korea; jungha@jbnu.ac.kr
[5] Department of Food and Life Science, Pukyong National University, Busan 48513, Republic of Korea; choijs@pknu.ac.kr
* Correspondence: jungha@jbnu.ac.kr (H.A.J.); choijs@pknu.ac.kr (J.S.C.);

Contents

Table S1. Experimental conditions for functional assays.

Table S2. Binding sites and docking scores of active compounds along with reference compounds in the targeted GPCRs.

Table S1. Experimental conditions for functional assays.

Target GPCR	Assay	Species / Tissue [a]	Stimulus	EC_{50} of stimulant	Incubation	Measured Component	Positive control	Detection Method
D_3 (*h*)	Agonist	Human / CHO-K1	None (control: 300 nM dopamine)	0.5 nM	30 min/37 °C	cAMP	Dopamine	HTRF
	Antagonist	Human / CHO-K1	Dopamine (10 nM)	-	30 min/37 °C	cAMP	(+)-Butaclamol	HTRF
D_4 (*h*)	Agonist	Human / CHO-K1	None (control: 10 µM dopamine)	5.6 nM	10 min/37 °C	cAMP	Dopamine	HTRF
	Antagonist	Human / CHO-K1	Dopamine (100 nM)	-	10 min/37 °C	cAMP	Clozapine	HTRF
M_1 (*h*)	Agonist	Human / CHO-K1	None (control: 100 nM ACh)	0.6 nM	RT	Intracellular [Ca^{2+}]	ACh	Fluorimetry
	Antagonist	Human / CHO-K1	ACh (10 nM)	-	RT	Intracellular [Ca^{2+}]	Pirenzepine	Fluorimetry
M_2 (*h*)	Agonist	Human / CHO-K1	None (control: 3 µM ACh)	70 nM	10 min/37 °C	cAMP	ACh	HTRF
	Antagonist	Human / CHO-K1	ACh (300 nM)	-	10 min/37 °C	cAMP	Methoctramine	HTRF
M_3 (*h*)	Agonist	Human / CHO-K1	None (control: 1 µM ACh)	20 nM	RT	Intracellular [Ca^{2+}]	ACh	Fluorimetry
	Antagonist	Human / CHO-K1	ACh (100 nM)	-	RT	Intracellular [Ca^{2+}]	4-DAMP	Fluorimetry
M_4 (*h*)	Agonist	Human / CHO-K1	None (control: 1 µM ACh)	26 nM	10 min/37 °C	cAMP	ACh	HTRF
	Antagonist	Human / CHO-K1	ACh (100 nM)	-	10 min/37 °C	cAMP	PD102807	HTRF
M_5 (*h*)	Agonist	Human / CHO-K1	None (control: 0.624 µM ACh)	2 nM	RT	Intracellular [Ca^{2+}]	ACh	Fluorimetry
	Antagonist	Human / CHO-K1	ACh (10 nM)	-	RT	Intracellular	Atropine	Fluorimetry

						$[Ca^{2+}]$		
CGRP (*h*)	Agonist	Human / CHO-K1	None (control: 10 nM hCGRPα)	0.14 nM	30 min/RT	cAMP	hCGRPα	HTRF
	Antagonist	Human / CHO-K1	hCGRPα (0.3 nM)	-	30 min/RT	cAMP	hCGRPα(8-37)	HTRF
V_{1a} (*h*)	Agonist	Human / CHO-K1	None (control: 1 μM AVP)	0.45 nM	RT	Intracellular $[Ca^{2+}]$	AVP	Fluorimetry
	Antagonist	Human / CHO-K1	AVP (10 nM)	-	RT	Intracellular $[Ca^{2+}]$	$[d(CH_2)_5,^1Tyr(Me)_2]$-AVP	Fluorimetry
5-HT_{1A} (*h*)	Agonist	Human / BA/F3	None (control: 2.5 μM serotonin)	1.9 nM	RT	Intracellular $[Ca^{2+}]$	Serotonin	Fluorimetry
	Antagonist	Human / BA/F3	Serotonin (30 nM)	-	RT	Intracellular $[Ca^{2+}]$	(S)-WAY-100635	Fluorimetry

[a] All products including cell-line and reagents were provided by Cerep (Celle L'Evescault, France).

HTRF: Homogeneous time resolved fluorescence; RT: room temperature; AVP: vasopressin; ACh: acetylcholine; 4-DAMP: 1,1-dimethyl-4-diphenylacetoxypiperidinium iodide; hCGRPα: human calcitonin gene-related peptide.

Table S2. Binding sites and docking scores of active compounds along with reference compounds in the targeted GPCRs.

Targets	Compounds	Binding energy (kcal/mol)	Residues			
			Salt bridge interaction	H-bond interaction	π-interaction	Alkyl interaction
*h*D$_3$R	Dopamine [a] (Agonist)	–5.84	Asp110	Val111, Thr115, Ser196	Phe346 (π-alkyl)	Val111, Cys114
	Icariside II	–7.49 (Pose 1)	–	Asp110, Ile183, Ser182, Gly94	Tyr373 (π-alkyl), Leu89 (π-alkyl)	Leu89, Val180, Val86
		–6.92 (Pose 2)	–	Asp110, Ser366, Leu89, Gly93	Thr369 (pi-sigma), Thr365 (pi-pi T-shaped), Phe106, His349, Val86 (π-alkyl)	Ile183, Val107
*h*M$_2$R	QNB [a,b] (Antagonist)	–10.95	Asp103	Asn404	Tyr104 (π-cation, π-π stacked), Tyr155 (π-π stacked), Trp400 (π-π T-shaped), Thr190 (amide-π stacked), Ala194, Ala191 (π-alkyl)	–
	Icaritin	–9.92 (Pose 1)	–	Tyr104, Tyr403, Ala191	Asp103 (π-anion), Cys429 (π-sulfur), Tyr104, Trp400, Tyr403 (π-π T-shaped), Tyr104, Trp155, Ala191, Ala194 (π-alkyl)	Ala194, Val111
		–7.72 (Pose 2)	–	Ile178, Asn419, Tyr104, Tyr403	Trp422 (π-π T-shaped), Tyr426 (π-π T-shaped), Tyr80, Tyr83, Trp99, Tyr426 (π-alkyl)	–

[a] Reference agonist or antagonist.

[b] (3R)-1-azabicyclo[2.2.2]oct-3-yl hydroxy(diphenyl)acetate.
